# Something’s missing from my education: Using a cross sectional survey to examine the needs and interest of Canadian medical students relating to their roles as teachers and educators

**Published:** 2017-06-30

**Authors:** Alim Nagji, Karen Leslie, Eric Wong, Doug Myhre, Meredith Young, Ming-Ka Chan

**Affiliations:** 1 Department of Family Medicine, McMaster University, Ontario, Canada; 2 Department of Pediatrics, University of Toronto, Ontario, Canada; 3 Department of Family Medicine, Schulich School of Medicine & Dentistry, Western University, Ontario, Canada; 4 Distributed Learning and Rural Initiatives, University of Calgary, Alberta, Canada; 5 Department of Medicine, McGill University, Quebec, Canada; 6 Centre for Medical Education, McGill University, Quebec, Canada; 7 Department of Pediatrics & Child Health, Rady Faculty of Health Sciences, University of Manitoba, Manitoba, Canada; 8 Canadian Association of Medical Education, Canada

## Abstract

**Background:**

Current theory in medical education emphasizes engaging learners as educators while tailoring teaching to their learning needs. However, little is known about learners’ perceptions of their proposed roles as teachers and educators.

**Methods:**

Canadian medical students were invited to complete an English language online questionnaire structured to include: teaching experience, participation and/or awareness of teacher development at their school and awareness and/or interest in further training in medical education. The survey was developed by the Canadian Association for Medical Education (CAME) Membership Subcommittee, and distributed via the Canadian Federation of Medical Students (CFMS) email list and the CAME twitter account in March 2014.

**Results:**

Of the 169 undergraduate medical student respondents, 36% (n=61) reported a lack of prior teaching experience and 45% (n=73) were unsure if their school provided teaching instruction. Overall, 91% (n=150) indicated that they planned to incorporate teaching or medical education into their future careers.

**Conclusion:**

While the majority of medical student respondents are expecting or planning to teach, most report not having access to adequate training through medical school. Further effort is necessary to support medical students as teachers to prepare them for increased teaching responsibilities as residents and to expose them to potential careers in medical education.

## Introduction

Learners are important stakeholders in the medical education “enterprise,” ranging from participating and contributing to organizations such as the Canadian Federation of Medical Students (CFMS) to playing a role in curriculum development (e.g. sitting on curriculum committees) at their medical school.^1^ Their roles as future teachers and educators are highlighted in the CanMEDS framework through the Scholar Role, which emphasizes the role of active teaching and the supervision of other learners.^2^–^4^ The Canadian Association for Medical Education (CAME, http://www.came-acem.ca/), an organization for medical educators, has identified the importance of supporting medical trainees at all levels in contributing to the future of medical education through participation in teaching, curriculum design, educational leadership and scholarship.^5^ In addition, the Future of Medical Education in Canada (FMEC) undergraduate and postgraduate medicine reports highlight the importance of developing clinical teachers and supportive learning environments for medical learners with a specific emphasis on faculty development and increased collaboration.^6^,^7^ Residents may spend up to a quarter of their work hours in teaching or supervising trainees,^8^,^9^ therefore it is important that resources are made available to support the development of these skills early in undergraduate medical education to ease the transition to the resident’s teaching role.^8^–^16^ With the growing expectation that medical students should teach, and continue to teach throughout residency and into practice, there is a need to provide support for the development of teaching skills throughout the continuum of medical education.^17^ The provision of teaching development in medical school provides timely access for individuals who will play a pivotal role in teaching throughout residency and beyond. This need for training specific to skills relating to teaching and education was echoed in an international survey of medical student representatives from 102 countries where “medical students do not feel sufficiently capable to involve themselves in curriculum governance and quality assurance.”^18^,^19^ Placing more emphasis on the provision of teaching skills at an earlier stage of medical training could enhance learners’ capabilities to participate in curricular activities.

A number of schools have begun to adopt the CanMEDS framework for medical student training, highlighting a move towards educational alignment across the continuum of medical education. While the transition may be more direct for the Medical Expert Role, the Scholar role poses some unique challenges as medical students are typically on the receiving end of education rather than having an active participatory role, and are expected to quickly adapt to a teaching role as they transition into residency. There is a growing body of literature regarding the skills required to be a teacher in the health professions, highlighting the importance of training learners to be teachers.^20^ Programs that promote the acquisition of knowledge, skills and attitudes to facilitate effective learning are now widely available, and although the majority are focused at the faculty level, with some programming for residents.^21^ To our knowledge, there are no published studies about teaching programs targeting medical students in Canada, nor the training needs of medical students in relation to teaching. Understanding the needs of medical students in this area of learning will allow for the development of programs and resources to better prepare and support them in relation to their roles as teachers and educators.

The purpose of this study was to explore the experiences and needs of Canadian medical learners with respect to their current and future roles as teachers and educators. Results from this study are intended to inform local, national and international efforts in developing training and support systems to help medical students acquire the knowledge, skills and attitudes to fulfill such teaching roles and to support collaboration between independent institutions.

## Methods

### Participants

Medical students and residents at Canadian institutions were eligible to participate; there were no exclusion criteria.

### Data collection

In an effort to reach as many of the over 2000 medical students and residents as possible, the survey link was emailed out to all medical students registered with the Canadian Federation of Medical Students (CFMS) between February and April 2014, who requested communiqués from CFMS which represented 227 students. The link was also emailed to registrants of the Canadian Association of Interns and Residents (CAIR) (now known as Resident Doctors of Canada or RDoC) in March 2014. One reminder was sent one month later. Survey links were also distributed via the College of Family Physicians of Canada postgraduate trainee email list and the CAME Twitter account. A request was made to send the survey link out through the Fédération médicale étudiante du Québec (FMEQ). Distribution of the survey through the medical schools’ contact lists directly was not permitted. A chance to win a gift card was provided as an incentive to complete the survey.

### Survey development

The 2013 CAME Membership committee, a group of leaders in medical education consisting of both faculty and trainee representatives from across the country developed the survey that included questions to probe:

1. Teaching experience prior to medical school including any specific training received;
2. Awareness of, and participation in, teacher training and/or education development and scholarship at their institutions; and
3. Awareness of, and interest in, future involvement in medical education activities, and interest in further training in medical education.

Items for inclusion in the survey were derived from discussions amongst members of the CAME Membership committee following a review of the literature. Items were reviewed iteratively with members of the committee to create a draft survey. The survey was then piloted with a small sample of trainees attending a CAME “Meet and Greet” event at the Canadian Conference on Medical Education in April 2013. Feedback from this group of participants was incorporated and the survey was refined and finalized. Ethics approval was received by the University of Manitoba Research Ethics Board HS16304 (H2013:162).

In addition to the survey questions, demographic data including age, gender, level of training, current school, prior education, and career specialty choice(s) were collected. The survey was hosted on Fluid Survey (http://fluidsurveys.com/about/) in English.

### Data analysis

Descriptive statistics and proportion of responses were calculated for each question.

## Results

### Participants

Of the 190 complete responses, 169 were medical students, 18 were residents and three identified as being in the MD/PHD stream. The original intent was to include residents; however, due to lack of a complete mailing list for all residents, it was not possible to ensure national distribution of the invitation to participate. Given this low response rate, a decision was made to exclude residents and MD/PHD stream students and to focus on the data from the 169 medical student responses to improve generalizability of responses for this group. The total number of individuals directly registered to receive the CFMS communique was 227. The response rate for medical students recruited directly through the communique, is therefore estimated to be, 73.5% (169/227). However, it is difficult to accurately estimate this rate given that the email may have also been resent via local institutional mailing lists and participation was also recruited via Twitter.

Of the 17 medical schools in Canada, 15 had at least one respondent. Two of the Canadian francophone medical schools had no respondents. The demographic data of the 169 medical student respondents are outlined in Table 1. Respondents did not have to answer every question.

### Previous teaching experience

Slightly over one-third of respondents (61/168) reported a lack of any teaching experience and 35.7% (60/168) indicated having had experience with non-academic/non-medical teaching, most commonly, coaching in sports (n=9) or music (n=9) and tutoring (n=16) (Table 2). Participants reported varying degrees of preparedness for teaching and 38.3% (23/60) of those participants who had prior teaching experience had not received any formal instruction on how to teach. Furthermore, even respondents who reported previously attending teaching seminars (17/169), 41.2% (7/17) of those received no specific training for their more recent responsibilities (Table 3). Findings followed a similar pattern for those who taught problem based learning (n=6) or in a clinical setting (n=9), where 66.7% received no prior training.

### Teacher training in medical school

Forty-five percent of respondents (73/161) were unsure as to whether their school provided teaching instruction or elective opportunities in teaching. A small proportion (3.7%, 6/161) reported they knew that their school provided formal training on teaching with a slightly higher proportion (9.3%, 15/161) reporting that their schools provided elective experiences in the areas of teaching and/or medical education.

### Perceptions and plans for incorporation of teaching/education roles in future careers

A small proportion of respondents (6/152) reported no interest whatsoever in clinical teaching, and 6.6% (10/152) had no interest in non-clinical teaching, while the majority (155/161) reported interest in teaching.

Ninety-percent (150/165) of respondents indicated that they planned to incorporate clinical teaching or some aspect of medical education into their future careers and 24.8% (41/165) of participants felt that they were knowledgeable about what opportunities existed for them to be involved in medical education in their future careers. Participants reported a desire to have access to this information, and suggested ways that this involvement might occur; suggestions included formal workshops, and opportunities to hear “stories” from faculty about their career paths that incorporated teaching and medical education. Only 12.4% (20/161) identified having a current mentor in medical education.

### Interest in additional training

Participants expressed a desire for additional learning in various areas, with the most interest in additional learning relating to clinical teaching (75%, 114/152), non-clinical teaching (51.3%, 78/152) and simulation based teaching (40%, 60/150). There was less interest for further training relating to the assessment of learning, program evaluation, research, leadership and policy work relating to medical education (Tables 4 and 5).

In the free text comment section, one participant shared a perspective that frames the data presented in the previous sections:

I am well aware that being an educator is a role of a physician. However, I have very minimal idea of what exactly that entails. It seems to be that residents and attending physicians are expected to just somehow figure out how to teach or innately know how when the time comes that they find themselves in the role of educator.

## Discussion

Findings from this study suggest that our respondents, drawn from most Canadian schools, have an interest in being more involved in the teaching and learning process; however currently perceive that they have little opportunity to access relevant training programs and supports.

Within the context of this study, the availability of teaching training programs is difficult to ascertain, as no central repository of such educational programs exists. Therefore, it is difficult to determine if our findings suggest a true lack of available training, or a lack of awareness amongst our participants about available programs, or that programs are only offered at a different stage of training (i.e., clinical years). It is possible that elective or informal programs are less well advertised than other programs within the formal medical school curriculum, or are simply still in their infancy. This area is a key theme for future collaboration in resource development and could be critical in improving access and availability to these programs.

Faculty development programs have been in place at all medical schools in Canada for a number of years.^22^ Some of these have been expanded to offer training to postgraduate and undergraduate trainees, and these student-as-teacher programs have been described in the medical education literature.^23^ The literature reports on a variety of models such as workshop-based content and longitudinal programs for residents-as-teacher training.^14^,^16^,^24^–^26^ Even so, a recent study of residents revealed that only 60.1% of respondents were aware of educational experiences within their own institution to support their development as teachers.^24^ Even in institutions that have begun offering programs, advertising, uptake and barriers to access may be concerns.^27^

There are a number of benefits to bringing teacher development into the UME training period. One such benefit may be that by learning about effective teaching and learning strategies and theories, trainees may become more aware of their own learning needs and how to enhance the effectiveness of their own education, setting the stage for a lifelong learning and growth.

Studies that have reviewed the status of students-as-teachers program in the US reveal that the majority of programs are for students in their senior years (3^rd^ and 4^th^).^26^,^28^ Development of training programs for the early years of medical school may be useful in preparing students as teachers prior to them entering clinical clerkships where they are likely to have increased opportunities to take on teaching roles. Experiences with teaching and learning initiated during undergraduate training could then be continued through residency; such experiences may enhance the quality of clinical teaching by residents with the ultimate goal of bolstering the pool of scholarly clinician-teachers and educators upon graduation. This kind of teaching-specific education throughout the medical education continuum could help meet the demand for physicians who are teachers throughout their professional life cycle in both the academic and community environments.^29^ Good teachers are of value not only to learners but also to patients, families and the community and there is evidence suggesting links between hospitals that train learners and enhanced patient care outcomes.^30^

Many students become involved in curriculum committees during their undergraduate training. If students were able to learn *about* teaching and learning early in their medical school training, their contributions to the curriculum as teachers/coaches and mentors to peers could be enhanced.^26^,^31^ Such opportunities might also promote interest in medical education as an academic stream for career development.^29^ As more student-as-teacher curricula emerge across schools, there is potential for cross-institutional collaboration to maximize the impact of existing programs.^6^ The formation of national working groups can assist with knowledge translation to ensure medical students across the country are being targeted appropriately. Once foundational programming is developed and implemented, residency and faculty level programming should be adapted to create incremental learning, graduated responsibility and continued support as medical students transition to resident teachers then clinician-teachers and educators.^2^

This study has some limitations. First, responses were limited to those students registered with CFMS who also subscribe to receive CFMS communiqués, or those who received the survey link via the CAME Twitter feed. Medical students and residents are difficult to access due to a lack of complete mailing list nationally and lack of processes to access or recruit through each school’s postgraduate and undergraduate office, which requires specific approval at each institution, and represents a logistical barrier. Centralized processes through national organizations such as the AFMC or the Royal College/College of Family Physicians of Canada for research recruitment that involves trainee participants may be beneficial. Either a centralized means of coordinating ethics processes or acceptance of another accredited university ethics approval would also enhance recruitment strategies in the future.

Some medical students can be accessed through the CFMS mailing list, however, it is unclear how many students are captured through this means as the list is not directly distributed to every medical student; in some institutions, the mail-out is redistributed by the CFMS representative to others in the class. The CFMS mailing list does not encompass all francophone schools and the lack of a French translation limited our ability to reach students at the Francophone schools. In addition, the use of social media as a recruitment strategy may bias the participants towards engaged learners who may already be aware of or involved with organizations like CAME or other educational organizations. Secondly, our participant pool had good representation across Canada despite being clustered in pre-clinical years. This clustering may affect the results as pre-clinical medical students may not be as familiar with programs offered through their schools in the clinical years. Finally, participants may be a biased group in that those who chose to complete the survey are already more interested in teaching and future careers in medical education. However, if they are the keenest about teaching and were not aware of programs available at their school, then this is problematic and could reflect a need to restructure programs to empower these students earlier.

### Conclusion

This study suggests the need for and interest of medical students in the development of student-as-teacher programs in Canada. Our respondents report a desire to learn about opportunities to acquire competencies as teachers during their medical training and are therefore important stakeholders in the development and promotion of students-as-teacher programs. There appears to be a lack of student awareness or availability of local supports and programs to enable them to gain skills and experiences as teachers and educators. Many schools may currently be piloting such programs and there may be a role for a national group to collate available curricula and resources, share curricular content and formats and develop best practices. Extending faculty and resident as teacher programs to medical students may also address this perceived or actual void.

There is an opportunity for national organizations for medical education to coordinate efforts within and across medical schools to support the continued growth and career development of future medical educators.

## Figures and Tables

**Table 1:** Gender & year of training & university affiliation

Gender	Number	Percentage
Male	56	33
Female	113	67

**Training Year**		

Year 1	53	31
Year 2	56	33
Year 3	27	16
Year 4	33	20

**University**	**Count**	**Percentage**

Dalhousie University	3	1.8
McGill University	6	3.6
McMaster University	20	11.8
Memorial University	22	13.0
Northern Ontario School of Medicine	6	3.6
Queen’s School of Medicine	8	4.7
University of Alberta	7	4.1
University of British Columbia	15	8.9
University of Calgary	6	3.6
University of Manitoba	13	7.7
University of Ottawa	14	8.3
University of Saskatchewan	11	6.5
University of Toronto	22	13.0
University of Western Ontario	15	8.9
Université de Montréal	0	0
Université de Sherbrooke	1	0.6
Université Laval Faculté	0	0

**Total Responses**	**169**	

**Table 2:** Teaching experience – “What teaching experience do you have?”

Response	Percentage	Count
No Teaching experience whatsoever	36.3	61
Non-university/college teaching (Please specify: e.g. primary/secondary education, first aid instruction, etc.)*	35.7	60
Seminar Leader	10.1	17
Problem-Based Learning (PBL) Tutor	3.0	5
Clinical Skills Tutor	6.0	10
Teaching Assistant (Facilitator)	25.0	42
Teaching Assistant (Marker)	22.0	37
Teaching Assistant (Lecturer)	7.7	13
Course Instructor	6.0	10
Curriculum Developer	7.7	13
Other (Please specify)**	4.8	8

* Non-University Teaching (Individuals listed more than one experience):
Tutor (n=16) Sports (n=9) Music (n=9) Primary education (n=4) Secondary education (n=5) Basic Life Support (n=2) Camp Instructor Medical College Admission Test Instructor

** Other (Individuals listed more than one experience)
Tutor (n=4) Camp Counselor Clinical skills for medical students ACLS/PALS Sewing Instructor Sports Instructor Science Centre Teacher

**Table 3:** Training/preparation received for teaching roles

	Leading Seminars (%, n)	Facilitating PBL (%, n)	Clinical teaching skills (%, n)	Being a teaching assistant (%, n)	Instructing a course (%, n)	Curriculum development (%, n)
None	41.2 (7)	66.7 (4)	66.7 (6)	59.2 (32)	44.4 (4)	53.8 (7)
Formal education: degree or diploma	0	0	11.1 (1)	0	22.2 (2)	7.7 (1)
Formal education: certificate	0	0	0	7.4 (4)	11.1 (1)	7.7 (1)
Formal education: (in class, non cert/dip/degree)	29.4 (5)	16.7 (1)	11.1 (1)	1.9 (1)	11.1 (1)	7.7 (1)
Online modules/learning	5.9 (1)	0	0	11.1 (6)	0	15.4 (2)
Workshop (s)	52.9 (9)	0	22.2 (2)	27.8 (15)	22.2 (2)	23.1 (3)
Other: Mentoring	5.9 (1)	16.7 (1)	0	1.9 (1)	0	7.7 (1)
Total responses*	17	6	9	54	9	13

* Respondents may have indicated more than one response

**Table 4:** Future career goals in medical education

	Strongly Disagree (%, n)	Disagree (%, n)	Neither Agree nor Disagree (%, n)	Agree (%, n)	Strongly Agree (%, n)	Total Responses
I plan on incorporating clinical teaching into my future career	1.8 (3)	0.6 (1)	6.7 (11)	44.8 (74)	46.1 (76)	165
I plan to incorporate some form of medical education into my future career	1.8 (3)	0.6 (1)	9.7 (16)	45.5 (75)	42.4 (70)	165
I feel informed about opportunities related to medical education in a medical career	6.1 (10)	35.8 (59)	33.3 (55)	20.6 (34)	4.2 (7)	165
I know what roles are available for physicians who work in medical education	5.5 (9)	34.5 (57)	32.7 (54)	24.2 (40)	3.0 (5)	165
I am aware of the kind of work that medical educators do	1.8 (3)	32.1 (53)	31.5 (52)	29.1 (48)	5.5 (9)	165

**Table 5:** Current reported interest in teaching

	No interest whatsoever (%, n)	Mild interest (%, n)	Moderate interest (%, n)	Strong interest (%, n)	Very strong interest (%, n)	Total Responses
Teaching in the clinical setting	3.9 (6)	2.0 (3)	19.1 (29)	44.7 (68)	30.3 (46)	152
Non-clinical teaching (e.g. tutorials, lectures, classroom based teaching, etc.)	6.6 (10)	13.8 (21)	28.3 (43)	34.2 (52)	17.1 (26)	152
Assessment and evaluation of learning	10.0 (15)	27.3 (41)	33.3 (50)	21.3 (32)	8.0 (12)	150
Technology enhanced learning (e.g. e-learning)	20.7 (31)	32.0 (48)	28.7 (43)	13.3 (20)	5.3 (8)	150
Simulation-based education	7.3 (11)	22.7 (34)	30.0 (45)	30.7 (46)	9.3 (14)	150
Educational Research	27.3 (41)	33.3 (50)	21.3 (32)	12.0 (18)	6.0 (9)	150
Educational Policy	34.9 (52)	27.5 (41)	21.5 (32)	10.1 (15)	6.0 (9)	149
Educational Administration	36.7 (55)	26.0 (39)	20.7 (31)	12.0 (18)	4.7 (7)	150
Program Evaluation (e.g. rotation or course evaluation, evaluation of an innovation, etc.)	20.1 (30)	28.9 (43)	25.5 (38)	19.5 (29)	6.0 (9)	149
Other (please describe)*	72.7 (48)	1.5 (1)	22.7 (15)	1.5 (1)	1.5 (1)	66

* Other (Individuals provided only two responses):
Interdisciplinary education Simulation
